# Nanobase.org: a repository for DNA and RNA nanostructures

**DOI:** 10.1093/nar/gkab1000

**Published:** 2021-11-08

**Authors:** Erik Poppleton, Aatmik Mallya, Swarup Dey, Joel Joseph, Petr Šulc

**Affiliations:** School of Molecular Sciences and Center for Molecular Design and Biomimetics, The Biodesign Institute, Arizona State University, 1001 South McAllister Avenue, Tempe, AZ 85281, USA; School of Molecular Sciences and Center for Molecular Design and Biomimetics, The Biodesign Institute, Arizona State University, 1001 South McAllister Avenue, Tempe, AZ 85281, USA; School of Molecular Sciences and Center for Molecular Design and Biomimetics, The Biodesign Institute, Arizona State University, 1001 South McAllister Avenue, Tempe, AZ 85281, USA; Wyss Institute, Harvard University, Boston, MA 02115, USA; School of Molecular Sciences and Center for Molecular Design and Biomimetics, The Biodesign Institute, Arizona State University, 1001 South McAllister Avenue, Tempe, AZ 85281, USA; School of Molecular Sciences and Center for Molecular Design and Biomimetics, The Biodesign Institute, Arizona State University, 1001 South McAllister Avenue, Tempe, AZ 85281, USA

## Abstract

We introduce a new online database of nucleic acid nanostructures for the field of DNA and RNA nanotechnology. The database implements an upload interface, searching and database browsing. Each deposited nanostructures includes an image of the nanostructure, design file, an optional 3D view, and additional metadata such as experimental data, protocol or literature reference. The database accepts nanostructures in any preferred format used by the uploader for the nanostructure design. We further provide a set of conversion tools that encourage design file conversion into common formats (oxDNA and PDB) that can be used for setting up simulations, interactive editing or 3D visualization. The aim of the repository is to provide to the DNA/RNA nanotechnology community a resource for sharing their designs for further reuse in other systems and also to function as an archive of the designs that have been achieved in the field so far. Nanobase.org is available at https://nanobase.org/.

## INTRODUCTION

The field of nucleic acid nanotechnology (1,2) is a rapidly expanding area of research in which DNA and RNA are designed to self-assemble into static and dynamic architectures with nanoscale precision. Since the inception of the field about forty years ago, DNA as well as RNA nanostructures of increasing size and complexity have been experimentally achieved (3,4). These structures have been used in wide range of applications including drug delivery (5), antivirals (6), immunotherapy (7), immunology (8), nanoelectronics (9,10) and photonics (11). To support the variety and complexity of DNA and RNA structures that researchers design, a number of nanostructure design tools (12–20) have been developed over the past 20 years. Despite having these human-readable and visualizable schematics, published papers often only include the sequences used to form the structure. While this information is sufficient for experimental reproduction of individual structures, it does not allow for extensive design, editing and repurposing of the structures, where higher levels of complexity and precision can be achieved through iterative design on existing structures and motifs. To encourage sharing of original design files among the various research labs, we present here Nanobase.org: a bionanotechnology database. The goal of the database is not only to create, for the first time, a public repository where researchers can share their design, but also to encourage adoption of conversion into file formats that can be easily further edited or used to initiate a molecular simulation for *in silico* characterization.

As DNA/RNA nanotechnology develops away from simple proof-of-concept structures into applications, it is imperative that the groups working on this are able to share and improve on existing designs to reduce the number of times the wheel has to be re-invented by various experimental groups. We hope that through sharing design parameters of nanostructures, the field can achieve higher yields of products, higher precision control of flexibility, and more multivalency in function. As experimentally realized designs have increased in complexity, using the coarse grained DNA and RNA model, oxDNA (21–25), has become a common addition to experimental realization of nanotechnology designs. Recent extension of the model also allows for a coarse-grained representation of proteins for DNA/protein or RNA/protein hybrid nanostructures (26). These simulations are often used to inform the design process prior to experiments as the model provides high-resolution data on inter-nucleotide distances, structure flexibility and duplex angles. They are also often used to rationalize results of experiments as the 2D images from AFM and TEM only provide a 2D projection of the structure, and the structures themselves may be affected by surface effects from adhering to the substrate (27), and the 3D class averages from cryo-EM provide higher resolution, but are inherently average structures and do not capture the dynamics of individual possible configurations (28). The one exception to this is some limited work done using tomography to study ensemble dynamics of 3D structures (29). Because most design tools either have native export to the oxDNA format or conversion is supported by the Tacoxdna webserver (30), oxDNA has become something of a *lingua franca* for DNA structure design, and currently is the only available format into which both DNA and RNA nanostructures can be exported into from a wide range of design tools. As such, in addition to original design files, the oxDNA format is a key part of the Nanobase server design.

Nanobase joins a collection of tools developed and maintained by our group and collaborators that have the common goal of improving data sharing and increasing the accessibility of simulations for DNA and RNA nanotechnology. There are four tools already published:

- A public webserver, Tacoxdna (30), which hosts a variety of conversion tools to convert designs into the oxDNA format.
- A simulation visualizer and structure editor, oxView (17), which simplifies simulations setup and trajectory visualization.
- A Python package for trajectory analysis, oxDNA Analysis Tools (17). This package provides pre-made scripts for a variety of common simulation analysis pipelines as well as utilities that support researchers own simulation analyses.
- A public webserver, oxdna.org (31), which facilitates modeling to experimentalists who may not be familiar with simulation techniques to run equilibrium sampling simulations.

In addition to the oxDNA-related tools maintained by our group, the DNA/RNA nanotechnology field has a rich suite of tools for the design and characterization of nanostructures. Design tools include:

- Tiamat (12), one of the first design tools, which is still popular with some groups. Though old, it has one of the most popular interfaces for free-form design of structures.
- CaDNAno (13), the most popular design tool in the field which is used to create schematics for DNA origami structures on either a square or honeycomb lattice.
- vHelix (14), A plugin for Maya and command line interface which converts polyhedral meshes into DNA origami wireframe structures with robust scaffold routing.
- Athena (15), A graphical interface which implements the older DAEDALUS, PERDIX and METIS command line interfaces for converting 3D wireframes into wireframe DNA origami in a more user-friendly interface.
- Adenita (16), a plugin for the broader molecular design tool SAMSON which integrates not only DNA and RNA design, but also protein and nanoparticles.
- oxView (17), the previously mentioned browser-based oxDNA visualization software is also a freeform design tool which can directly edit simulation files.
- Scadnano (18), an updated, browser-based CaDNAno interface with an improved scripting interface for algorithmic development of nanostructures.
- MagicDNA (19), a MatLab executable which automatically handles scaffold routing and staple generation for large, 3D, multi-scaffold lattice-based DNA origami structures.
- ENS Nano (20), a tool which integrates design of non-parallel lattice-based structures and simple simulation methods for optimization of crossover placement.

There are also other structure characterization and simulation methods built for the DNA nanotechnology community. These include:

- CanDo (32), a finite element analysis method which can take in CaDNAno or Tiamat designs and predict the equilibrium structure and flexibility.
- mrDNA (33), a multiscale molecular dynamics package which rapidly produces equilibrium atomistic structures of DNA origamis via simulation at multiple resolutions.
- SNUPI (34), a newer finite element analysis method which improves upon the CanDo tool with more accurate handling of single and double-stranded DNA.

Taken together, these tools provide a robust computational environment for the design of, especially, DNA nanostructures. However, a major gap in the existing tool environment is a way to share designs for further iteration and analysis. This is where Nanobase comes in; we hope that by creating a central repository for sharing design ideas, better comparisons between designs can be made and existing designs can be further optimized and re-used.

At the time of writing, Nanobase contains 57 unique structures that were provided by experimentalists working in the field, as well as structures that have been provided by theoreticians working on the designs. Each uploaded nanostructures includes a link to the publication where it is characterized. Furthermore, for all the structures that were deposited so far, the original design file from the design tool is provided, along with its conversion into oxDNA format as well as PDB format. Once the structure is converted into oxDNA format, further conversion into PDB format can be easily realized via Tacoxdna server. While we encourage users to convert and upload the design also in oxDNA format, it is not necessary. Our group is however committed to manually convert all uploaded structures in the database into oxDNA format to ease sharing between different groups, as well as to allow for the structure to be able to harness the ecosystem of tools available for oxDNA format, including interactive editing in oxView or computational modeling via oxDNA.org.

Nanobase is designed with a user friendly GUI interface, both to submit one’s own designs as well as to search the repository for deposited structures. We hope that this database is a resource that improves collaboration between researchers and brings greater innovation to the field.

## DATABASE USE AND ACCESS

### Browsing the database

**Figure 1.**
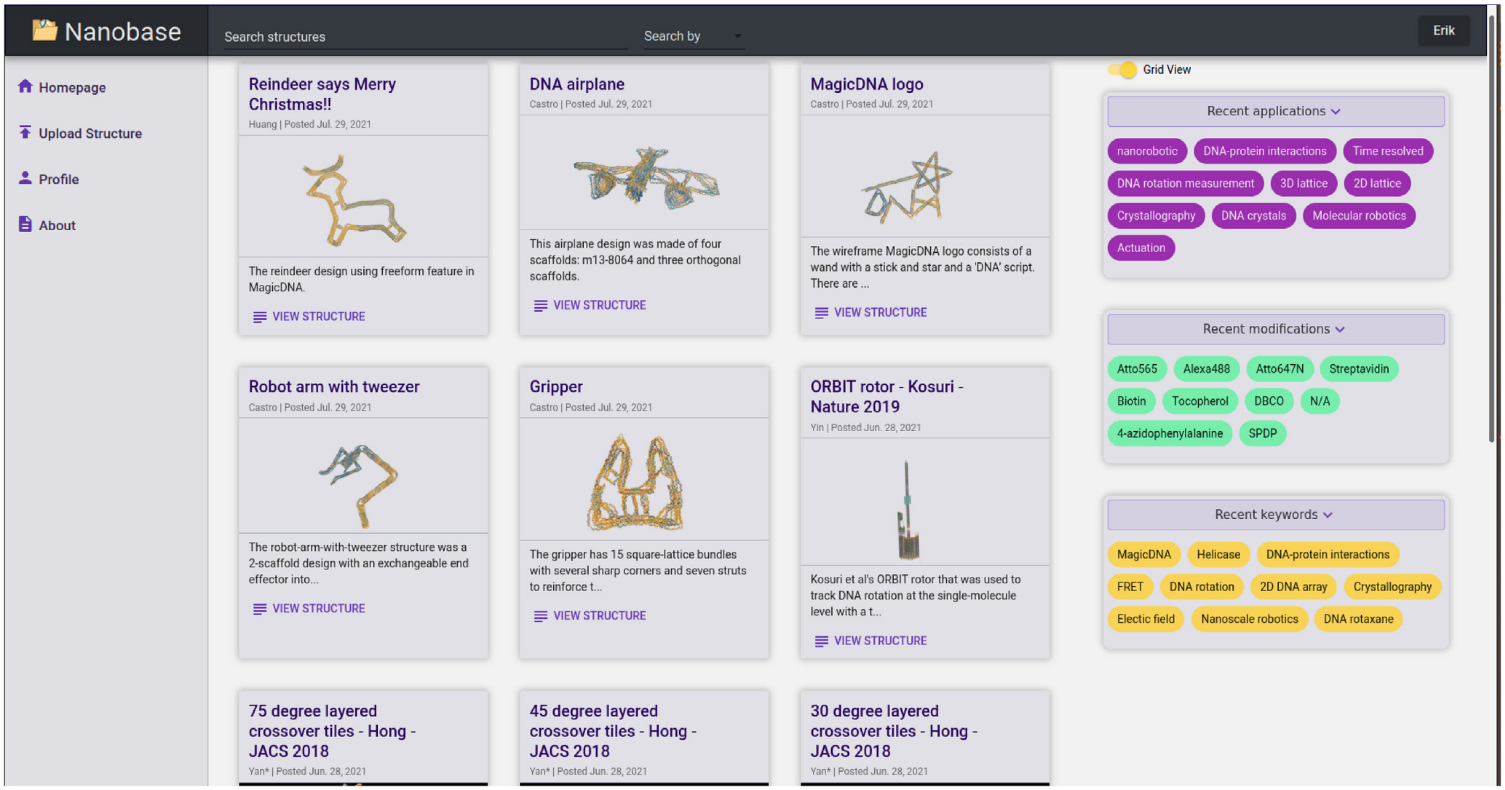
The nanobase home page in grid view. On the left is the navigation bar, the center column contains links with images and descriptions to database entries and on the right are sorting options which allow users to filter the structures displayed in the center column. The top bar contains search options.

**Figure 2.**
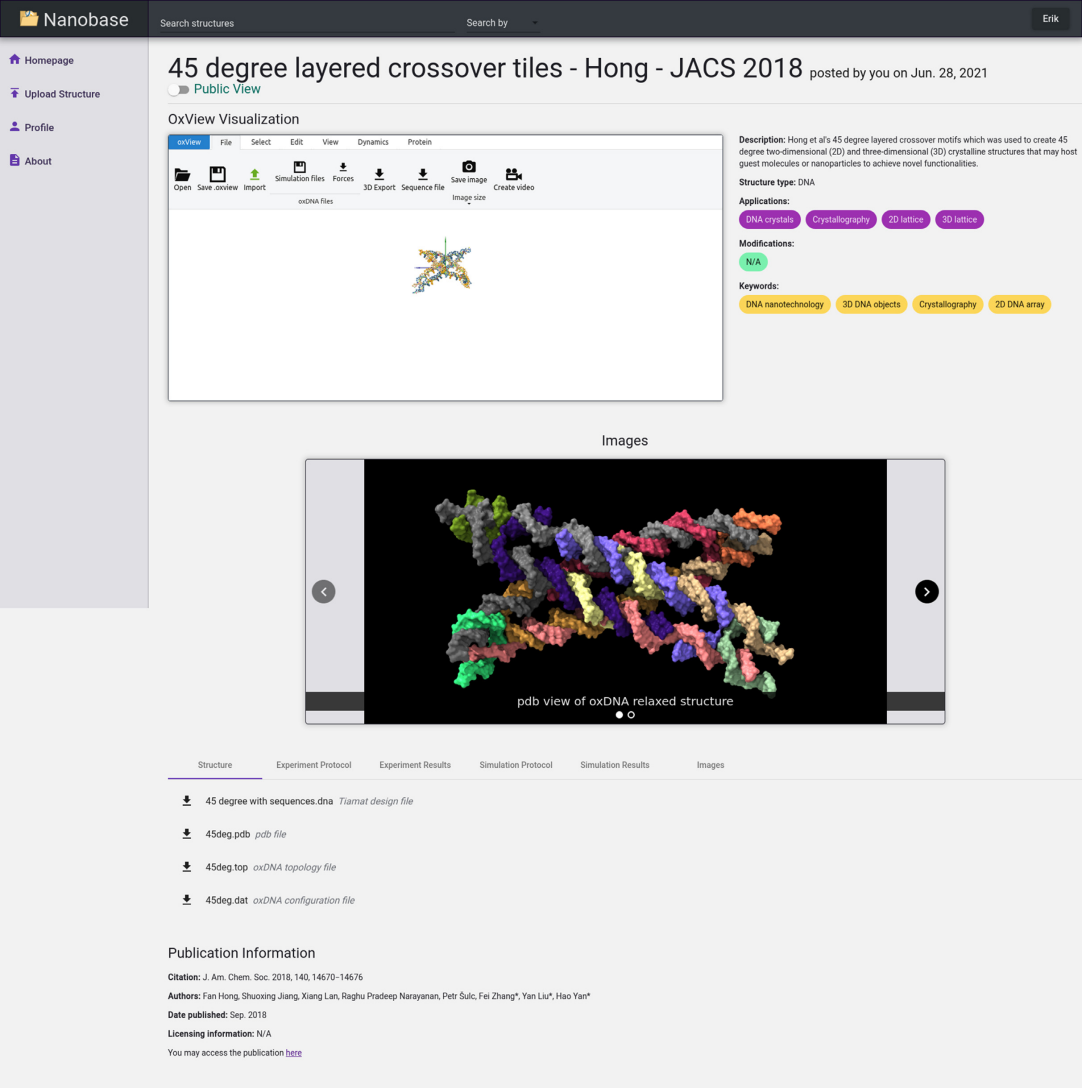
An example of a structure page. In this case, the 45° crossover unit from Ref. (35). The top half of the page shows a relaxed oxDNA configuration in an oxView iframe and the description and tags associated with the structure. Below that is the gallery of images included by the uploader. The bottom of the page contains structure information where original design files and oxDNA structure files can be downloaded as well as publication information.

The database is freely available at nanobase.org, and is compatible with all major browsers (Chrome, Firefox, Safari, Edge) on all major operating systems (Windows, Linux, Mac OS). The database main landing page (Figure 1) is a view containing all the structures deposited in an infinite scroll and one can browse all structures by scrolling down. It is possible to switch the main page view from ‘List View’, where structures are shown in a single column and you can browse them by scrolling down, to ‘Grid View’, where structures are shown in multiple columns (Figure 1). There are also various options for filtering the shown structures. On the sidebar, users can select tags to limit the current display to only contain structures that were tagged with specific keywords, applications or modifications. There is also a search bar where users can search for structures via title, paper author, application, modification, keyword, or uploader. The system uses ElasticSearch (36), so typos are tolerated in search parameters. On each structure’s page, all the information from the upload forms is displayed (Figure 2). This includes the brief description and the tags. There is also an interactive oxView frame where the structure is displayed in 3D if an oxDNA configuration/topology was provided, and an image gallery where the images uploaded by the submitter are displayed. Below the image gallery are download links for all the uploaded files, categorized by type. The first tab is for structure designs followed by statistics on the design scraped from oxDNA files, if provided, and the following tabs include files categorized as experimental protocols, experimental results, simulation protocols, simulation results, and images. Below the structure information is publication and licensing information. Here you will find the citation and link to the original publication, plus any licensing and patent information, if provided.

In the left sidebar, there is the option to download the database under ‘Quick Download’. This allows the user to download a zip file containing either/both the backend database or all structure files. The zip files are quite large and so are only updated on a weekly basis. We hope that this option will be useful for users interested in collecting data on a wide arrangement of DNA/RNA nanotechnology designs for reviews and comparative studies.

### Depositing a structure

To deposit a structure, users must first create an account on the server. This both helps prevent spam through email verification and also gives the maintainers a point of contact if followup on a deposited structure is required. Once an account is created, the user can submit a structure by selecting ‘Upload Structure’ in the sidebar. On this page, the user will be taken through a series of forms to fill out in order to populate all the database fields. The first form is structure information. Here the uploader provides a name for the structure as well as tags for keywords, applications, and modifications which are used as search parameters. The user also specifies what type of molecule this is (DNA, RNA, DNA/RNA hybrid, nucleic acid–protein hybrid, or other) and a brief description.

The second page is publication information. The simplest way to add a publication is through a DOI search. The uploader may paste the DOI of their manuscript into the DOI box and click ‘Autofill information’. This will query the Crossref API and autofill information for most publications. Uploaders can also enter publication information manually if the manuscript information is incorrect or not indexed in Crossref. On this page, it is also possible to note if the structure is covered under any licensing or patent restrictions.

The third page is design and protocol information. The first field is for design files. The most important part of this page is to include the original design file to facilitate the sharing of techniques and motifs among the community. In addition to uploading the original structure design file, it is highly recommended to also export the original structure file to the oxDNA format to allow 3D visualization of the submitted structure, using provided tools at oxView.org or Tacoxdna servers. On this page, we also provide space to upload experimental and simulation protocols and results. These are optional, however including them will improve the utility and readability of the database. Finally, there is space to upload images. These are displayed as a gallery on the structure information page and the uploader can select one image to use as the lead image in the browse and search pages. This image should be a representative image of the structure and can come from experiments or simulations. If the user provided the oxDNA format, an interactive 3D view of the structure in embedded oxView window will be available for the users that view the structure in the database. For hybrid nanostructures that also contain a protein, the oxView visualization tool also supports extended oxDNA format that is used by the ANM-oxDNA model and represents protein at residue level. The oxView tool can load both protein and DNA/RNA nanostructure and export to the ANM-oxDNA format (26), which can then be uploaded to the database.

As the final step of the upload, the user has the option to select that the uploaded structure will be initially private and available via the home page and search only after a specified date. The structure will be uploaded and the user will be able to share it using a unique link that will be generated at the time of upload.

## SYSTEM DESIGN AND IMPLEMENTATION

Since Nanobase is a repository of designed structures, the key features of it are that it is easy for researchers to deposit designs and easy for users to search for designs across a variety of parameters. The database backend is implemented in MySQL to make it easy to query the database not only by name, but also by Application, Author, Keywords and Modifications. Search is performed using ElasticSearch, which also helps submitters re-use keywords, applications and modifications through autocomplete. The website uses a Flask (Python3) and Angular 11 (TypeScript) stack to dynamically display pages. Embedded in each structure view page is an iFrame running an instance of oxView which provides 3D visualization of each structure in the database. The software used to serve the web application includes Nginx and Gunicorn. The source code of the database is available as a free software under GNU Public License at https://github.com/sulcgroup/nanobase.

## DISCUSSION

Nanobase represents the first database specifically tailored to the DNA and RNA nanotechnology communities. Previous nanotechnology databases such as Pubvinas (39), Nanowerk and NanoMaterialsSummaries have focused broadly on nanotechnology and the experimental results of studies but contain very few DNA/RNA structures and are more focused on characterization than design. Nanobase’s focus on nucleic acid nanotechnology and sharing design schematics is a unique niche which has so far not been addressed by the research community. With sufficient community buy-in, we hope that Nanobase can become something like the PDB (40) for nucleic acid nanotechnology: the first stop for researchers in the field when searching for previously realized structures. We hope that having a one-stop repository for schematics both allows for re-use of structures and inspires future innovation either by editing an existing structure or by data-mining the database for parameters that make for successful structures. The current set of structures deposited in Nanobase, collected and curated from various experimental groups, represents the largest repository of DNA and RNA structures available.

**Figure 3.**
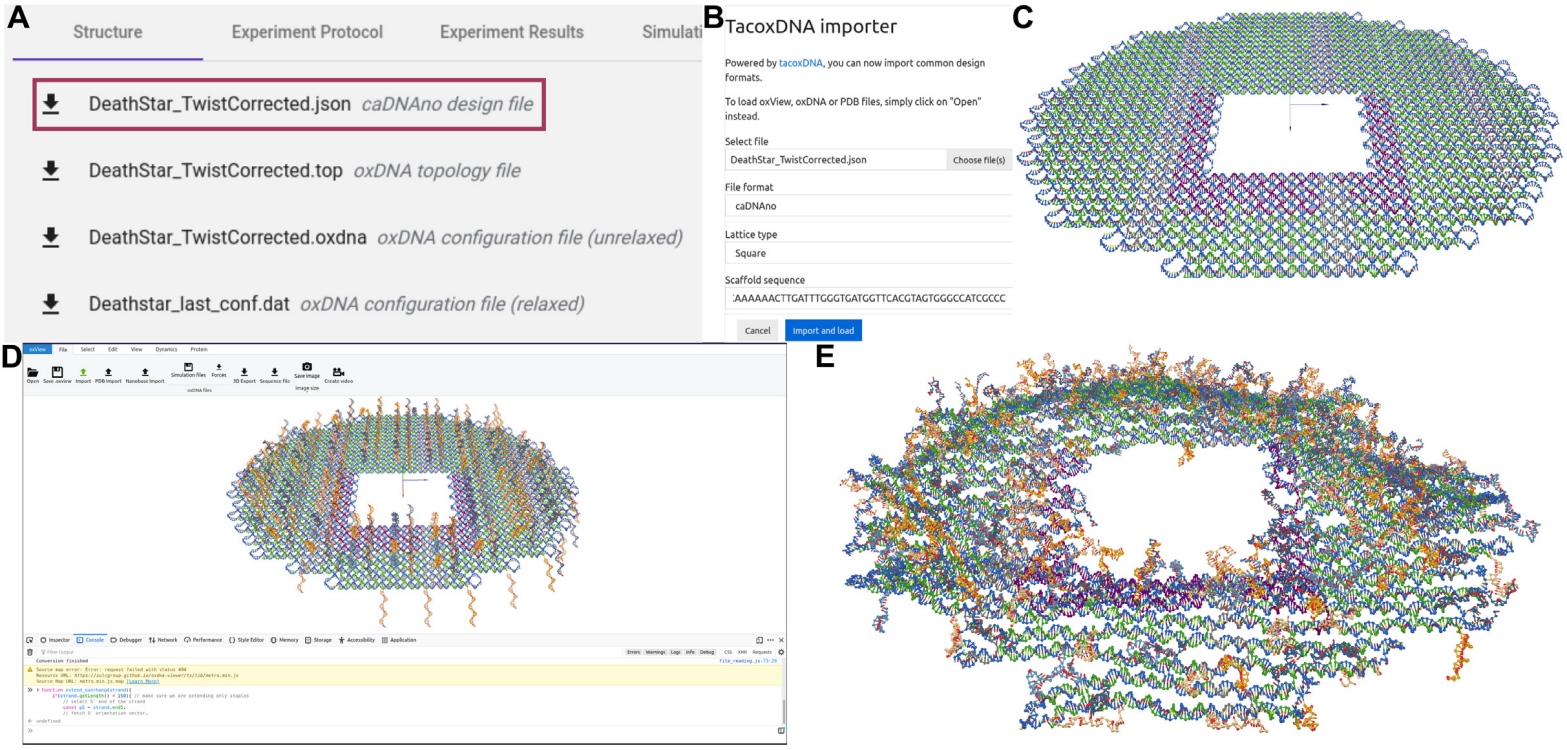
An example of using an entry from Nanobase to set up an oxDNA simulation and get a full staple list. In (37), the authors use single stranded extensions to control the landing orientation of 2D DNA origami on a surface. Their Cadnano file, however, does not contain these overhangs, presumably because it was easier to add them to the staple list than manually extend each strand in the Cadnano interface. Here, we demonstrate a workflow from (38) for taking the original design file from Nanobase (**A**), importing it into oxView (**B**, **C**), using the scripting interface to add the 20T single stranded extensions described in the paper (**D**), and relaxing the structure for oxDNA simulation (**E**). This workflow takes only a few minutes to prepare for simulation and could be used to test the properties of many different modifications in parallel through simulation.

As the field of nucleic acid nanotechnology steps into adolescence, the need of a streamlined approach becomes critical to ensure exponential growth while maintaining reproducibility of the variety of design paradigms for creating self-assembled DNA and RNA nanostructures. Nanobase.org is a perfect tool in this juncture that will enable sharing of the design techniques and schematics for previously successful designs. This will promote the development of the field through more re-use of existing motifs and iteration on designs. It can be particularly useful with regards, but not limited to, the following aspects:

- When a research group wants to use a partial feature of a particular nanostructure design, it will be much more advantageous to start from the original design file and just make the necessary changes as required.
- We envision that Nanobase.org will particularly be useful for the non-expert researchers without having to start a design from scratch. This will catalyze the expansion of the field at a even rapid pace.
- We envision that a database like this will inspire comparative studies, both experimental as well as theoretical, across higher sample size of nanostructures. For example - dependence of properties such as serum stability (41), cell penetration efficiency (42), biodistribution (43) etc. can be studied over higher sample size with the help of a database like this. More fundamental questions such as dependence of thermodynamic and kinetic properties of DNA nanostructures can also be studied as a function of scaffold sequence, shape or structural density etc.
- With simulations becoming more popular in the field, it is becoming easier to test many design iterations on the same structure *in silico* prior to experimental realization (Figure 3). We hope that by converting structures deposited into Nanobase into the oxDNA format, we can facilitate larger scale simulation studies of the design parameters that affect structure assembly, flexibility and function.

We encourage the community to use this opportunity to share their expertise and by sharing their designs, create a repository that will support future researchers in learning design techniques, build on previous experimental results, and grow the potential applications of the field.
